# Early Prediction of Acute Kidney Injury Using the Furosemide Stress Test in Pediatric Cardiac Surgery Patients

**DOI:** 10.3390/children13030358

**Published:** 2026-02-28

**Authors:** Ömer Özden, Murat Tanyildiz, Aslı Ece Yakici, Ezgi Nur Alper, Mete Han Kızılkaya, Mehmet Biçer, Cemile Pehlivanoğlu, Ender Ödemiş, Atıf Akçevin

**Affiliations:** 1 Pediatric Intensive Care Unit, Koç University Hospital, Istanbul 34025, Türkiye; mtanyildiz@kuh.ku.edu.tr (M.T.); ayakici@kuh.ku.edu.tr (A.E.Y.); ealper20@ku.edu.tr (E.N.A.); 2 Pediatric Cardiology Unit, Koç University Hospital, Istanbul 34025, Türkiye; metkizilkaya@kuh.ku.edu.tr (M.H.K.); eodemis@kuh.ku.edu.tr (E.Ö.); 3 Pediatric Cardiovascular Surgery Unit, Koç University Hospital, Istanbul 34025, Türkiye; mbicer@kuh.ku.edu.tr (M.B.); aakcevin@kuh.ku.edu.tr (A.A.); 4 Pediatric Nephrology Unit, Koç University Hospital, Istanbul 34025, Türkiye; cpehlivanoglu@kuh.ku.edu.tr

**Keywords:** congenital heart disease (CHD), furosemide stress test (FST), acute kidney injury (AKI)

## Abstract

**Highlights:**

**What are the main findings?**

- The furosemide stress test enabled earlier prediction of acute kidney injury risk than KDIGO criteria after pediatric cardiac surgery.
- Reduced urinary response to the furosemide stress test was independently associated with acute kidney injury.

**What are the implications of the main findings?**

- The furosemide stress test may serve as a simple bedside tool for early risk stratification.
- Early identification of high-risk patients may support closer monitoring and timely nephroprotective interventions.

**Abstract:**

Introduction: We evaluated the furosemide stress test as an early predictor of acute kidney injury following pediatric cardiac surgery, in comparison with the Kidney Disease: Improving Global Outcomes (KDIGO) diagnostic criteria. Materials and Methods: This single-centre retrospective study evaluated pediatric patients who underwent open cardiac surgery between March 2019 and September 2024 at Koç University Hospital. The evaluation included pre-operative, intra-operative, and postoperative variables; a two-hour assessment of urinary response to the first dose of furosemide upon admission to the intensive care unit; and rates of acute kidney injury. Results: A total of 254 patients were included, and 53 patients (20.8%) developed acute kidney injury according to KDIGO criteria. The mean furosemide stress test response was 9.86 ± 5.84 (median: 9.10) mL/kg/h in the non-AKI group and was significantly lower in the AKI group at 5.07 ± 4.73 (median: 3.33) mL/kg/h (*p* < 0.001). Receiver operating characteristic analysis demonstrated that the furosemide stress test has discriminative ability to predict acute kidney injury. The cut-off value was 6.104 mL/kg/h, and patients with a lower response had a higher risk of developing acute kidney injury. Sensitivity and specificity were 69.8% and 69.7%, respectively. Acute kidney injury was diagnosed at a median of 18 h using KDIGO criteria, whereas the furosemide stress test enabled earlier prediction of acute kidney injury risk at a median of 5 h. Conclusions: The findings support the potential clinical utility of the furosemide stress test in the early stages after pediatric cardiac surgery to predict acute kidney injury.

## 1. Introduction

Acute kidney injury is commonly seen in critically ill neonatal and pediatric patients. Epidemiological data from large-scale studies have reported a range of 26% to 30% for the incidence of acute kidney injury within the first 7 days of admission to the pediatric intensive care unit (ICU) [1,2,3]. Acute kidney injury is more prevalent in pediatric patients undergoing cardiac surgery, with an incidence ranging from 27% to 52% [3,4,5,6]. Postoperative acute kidney injury has been associated with prolonged mechanical ventilation, extended stays in the ICU and hospital, and an increase in morbidity and mortality rates [7].

Currently, the diagnosis and classification of acute kidney injury are done using the “Kidney Disease: Improving Global Outcomes” criteria [8], which are based on an increase in serum creatinine levels or a decrease in urine output. Diagnosis and treatment options for acute kidney injury remain limited. Importantly, the diagnosis of acute kidney injury is often recognized at a relatively advanced stage using these criteria. Because of the limited efficacy and reliability of the criteria to detect early kidney injury and predict the severity of damage, it often results in delayed recognition of acute kidney injury and potentially contributes to a poorer prognosis for the pediatric patients in the ICU. Therefore, numerous biomarkers are currently under investigation to accurately predict and detect acute kidney injury in critically ill patients at an early stage [9], but they have not yet been adopted for routine use due to their high cost and practical limitations.

It has been suggested that acute kidney injury could be diagnosed using functional tests of the kidneys [9]. Furosemide is a readily available, cost-efficient loop diuretic medication that is used to evaluate renal tubular function. The urinary response to furosemide is decreased in patients with acute kidney injury due to a functional decline in renal tubules. The urine output of patients who have been given furosemide can be used as a reliable marker of renal tubular function. The furosemide stress test was first introduced to assess the renal function of adult patients in critical condition in 2013 [10]. The furosemide stress test allows for the standardized evaluation of renal function and urine output, and the result is described as the urinary output within the first 2 h after the administration of furosemide. Studies involving adult patients have reported a cut-off value for a normal furosemide stress test response as a urinary output higher than 200 mL/2 h [10]. However, the cut-off value for pediatric patients has not yet been clearly defined. Several studies have reported that the furosemide stress test outperforms serum and urine biomarkers in predicting progressive acute kidney injury [9]. Worldwide, furosemide is routinely used as a medication after cardiac surgery. Additionally, fluid intake and urinary output of patients in the ICU are closely monitored, so the furosemide stress test can be easily and widely used due to its cost efficiency, accessibility, and most importantly, reliability.

The use of the furosemide stress test after cardiac surgery may fulfil several characteristics of an ideal biomarker for acute kidney injury, as it provides an accurate assessment, is easy to apply, inexpensive, non-invasive, and reproducible [11]. Intravenous furosemide treatment is usually initiated within the first 24 h for pediatric patients who undergo cardiac surgery to decrease fluid overload or cardiac afterload once hemodynamic stabilization is achieved [12]. As the incidence of acute kidney injury is known to be higher among pediatric patients undergoing cardiac surgery, intravenous furosemide is commonly administered in this patient population during the early postoperative period. Nevertheless, very few studies have described the response to the furosemide stress test for predicting the progression of acute kidney injury in pediatric patients undergoing cardiac surgery.

We hypothesize that the furosemide stress test can be conducted during the early postoperative period and used to predict and detect acute kidney injury among pediatric patients undergoing cardiac surgery. This could enable early detection and intervention, allow for early treatment through comprehensive evaluation, and prevent the progression of acute kidney injury with appropriate management. If the development of acute kidney injury is identified in a timely manner based on the furosemide stress test response, clinicians can intervene immediately, decrease exposure to nephrotoxic medications, prevent hemodynamic instability in those with especially high risk for acute kidney injury, and provide the opportunity for closer follow-up [13,14].

## 2. Materials and Methods

This single-centre retrospective study evaluated the early predictive value of the furosemide stress test in detecting acute kidney injury among pediatric patients undergoing cardiac surgery requiring cardiopulmonary bypass. All surgeries in our hospital are conducted by two different pediatric cardiac surgeons. Postoperative data of patients aged 1 month to 18 years who underwent open cardiac surgery at Koç University Hospital during the time period of March 2019 to September 2024 were evaluated retrospectively.

The inclusion criteria were an age of 1 month to 18 years and a history of open cardiac surgery requiring cardiopulmonary bypass due to underlying congenital heart disease. The exclusion criteria were pre-existing renal insufficiency prior to the surgery, prior dialysis, renal transplant, being administered furosemide before or within 2 h of the first dose of the bolus furosemide infusion, and peritoneal dialysis prior to the first dose of furosemide. All data were collected retrospectively from electronic patient medical records and the clinical database of the cardiac ICU.

### 2.1. Data Collection

Previously recorded pre-operative, intra-operative, and postoperative patient data were evaluated in this study. The pre-operative data included demographic characteristics of the patients (age, gender, and body mass index), pre-operative creatinine and albumin levels, and surgical complexity scores, which were evaluated. The baseline creatinine and albumin levels of the patients were accepted as the levels measured within 48 h before surgery. Surgical complexity was evaluated in patients younger than 18 years of age who underwent cardiac surgery due to underlying congenital heart disease using the Risk Adjustment for Congenital Heart Surgery-1 (RACHS-1) score [15].

Intra-operative data included the cardiopulmonary bypass time, aortic cross-clamp time, and creatinine and lactate levels at admission to the ICU. Postoperative data included hourly urinary outputs. Urinary response to furosemide was considered the 2-h urine output following the first postoperative administration of furosemide, which was recorded both as the total amount and in relation to the patients’ weight (mL/kg/h). The timing of the first dose (hours postoperatively) and the dosage (mL/kg) were noted.

Furthermore, systolic blood pressure, central venous pressure, hemoglobin, albumin, and lactate levels were evaluated to assess the hemodynamics of the patients prior to the first dose of furosemide. Lactate, creatinine, albumin, and haemoglobin levels were monitored daily during their stay in the ICU. The duration of mechanical ventilation, stay in the ICU, daily fluid balance, hospital stay, and 28-day mortality rates were recorded for the patients.

Patients with mean arterial pressures two standard deviations below the normal range according to their age groups were considered hypotensive, and the inotropic agents that they were administered were recorded. The maximum vasoactive inotrope scores of all patients during the first 48 h were calculated and analyzed [16]. Renal replacement therapy (peritoneal dialysis, haemodialysis and continuous renal replacement therapy) and other extracorporeal procedures (plasmapheresis and extracorporeal membrane oxygenation) were noted.

The presence of acute kidney injury was evaluated according to the “Kidney Disease: Improving Global Outcomes” criteria and was staged as 1–3 depending on serum creatinine levels or changes in urine output [8]. For patients with acute kidney injury detected by the criteria, we evaluated the criteria stage and fluid balance on day 1 postoperatively, as well as the duration of acute kidney injury, nephrotoxic medication use, time of discharge, and criteria stage at 6–12 months postoperatively. Nephrotoxic medications were listed as vancomycin, piperacillin–tazobactam, aminoglycosides, colistin, bactrim, acyclovir, valgancyclovir, amphotericin-B, contrast agents, ACE inhibitors, and NSAIDs. The number of nephrotoxic medications that were used during the period of acute kidney injury development was noted. Stages of acute kidney injury and the number of nephrotoxic medications used were compared.

In our clinic, the dosage and timing of the furosemide bolus administration are clinical decisions made by pediatric intensive care specialists. As this study had a retrospective design, the dose of furosemide was not predetermined or standardized within a research protocol. However, in our routine clinical practice, the initial furosemide dose is generally administered as 1–2 mg/kg per dose (maximum: 40 mg/dose). The observed variability in dosing within the study population is related to differences in patients’ body weights and the application of the maximum dose limit of 40 mg. Therefore, in some patients, the calculated dose per kilogram appeared lower due to this upper dose restriction. All investigated patients were administered furosemide in the first 24 h post-surgery. Furthermore, daily fluid balance is determined as the difference between all fluids administered (including intravenous, nasogastric, oral, and blood products) and all outputs (urine, nasogastric, and drain outputs) from 7:00 in the morning until 07:00 of the following morning. The fluid balance was recorded in mL on the first day postoperatively for all patients and considered as negative, positive, or balanced. Since the body weights and admission times to the ICU of the patients varied, a standardized fluid balance for the first day was calculated for each patient separately.

The standardized fluid balance was obtained by dividing the daily fluid balance by the time from ICU admission to 07:00 the next morning (in hours) and the patient’s body weight (in kg). The resulting value was recorded in mL/kg/h and considered as negative, positive, or balanced. For all patients that were included in the study, their worst parameters in the first 24 h of admission to the ICU were used to assess and predict mortality risk based on the severity of their organ dysfunction using the Pediatric Mortality Risk-3 (PRISM-III) and Pediatric Logistic Organ Dysfunction-2 (PELOD-2) scores [17,18]. This study was approved by the Koç University Ethics Committee on Human Research (approval number: 2025.144.IRB2.062).

### 2.2. Statistical Analyses

Data were analyzed using SPSS (version 23.0; IBM Corp., Armonk, NY, USA) and R statistical software (version 4.3.1; R Foundation for Statistical Computing, Vienna, Austria) within RStudio (https://posit.co/ (accessed on 13 February 2026)) (Posit Software, Boston, MA, USA). Descriptive statistics (frequency, percentage, mean, standard deviation, minimum, maximum, and median) were applied to evaluate socio-demographic data and metric measurements in this study. Frequencies were represented as percentages (%). The chi-squared independence test was conducted to analyze categorical data.

In intergroup analyses, the Kruskal–Wallis test was used to compare metric values among the three groups, while the Mann–Whitney U test was used for comparisons between two groups. All statistical analyses were conducted using R statistical software (version 4.3.1; R Foundation for Statistical Computing, Vienna, Austria) within RStudio (Posit Software, Boston, MA, USA). Finally, receiver operating characteristic (ROC) curve analysis was performed to predict the development of acute kidney injury based on the standardized response variable. Finally, since the development of acute kidney injury (AKI) was defined as a binary outcome (present/absent), multivariable logistic regression analysis was performed to identify independent risk factors associated with AKI. Multiple logistic regression is a widely used method for evaluating the effect of independent variables on the probability of an event when the dependent variable is dichotomous. Model results were reported as regression coefficients (β), standard errors, z statistics, *p* values, odds ratios (ORs), and 95% confidence intervals (CIs). A two-tailed *p*-value of <0.05 was considered statistically significant in all analyses.

## 3. Results

**Figure A1:**
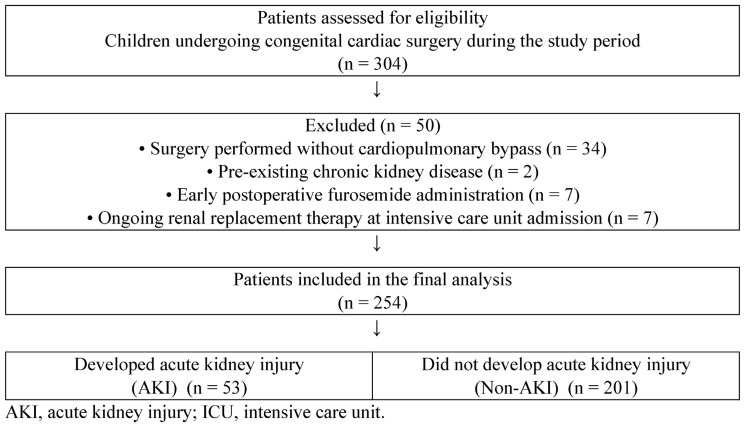
Study flow diagram.

This study evaluated 304 pediatric patients aged 1 month to 18 years who underwent cardiac surgery between March 2019 and September 2024. We excluded 34 patients who did not require cardiopulmonary bypass during the surgery, 7 patients who were admitted to the ICU with ongoing renal replacement therapy, 7 patients who were given another dose of furosemide within 2 h of the first bolus of furosemide, and 2 patients with pre-operative renal insufficiency. In total, 254 patients were included. Fifty-three of the patients (20.8%) developed acute kidney injury based on the “Kidney Disease: Improving Global Outcomes” criteria (Figure A1). The demographic characteristics of the patients included in this study are listed in Table 1.

We evaluated patients with acute kidney injury and without acute kidney injury in terms of demographic information, intra-operative and postoperative variables, scores (PELOD, PRISM, RACHS-1, and vasoactive–inotropic score), timing and dosage of the first furosemide administration (mg/kg), the first 2-h of urinary response after furosemide administration, standardized fluid balance on the day of admission to the ICU, duration of ICU stay, hospital stay and mortality rates (Table 2). Sex, body mass index, and pre-operative creatinine levels did not exhibit significant differences, but the risk of developing acute kidney injury was higher with younger age (*p* < 0.001). Furthermore, a significant rise in acute kidney injury development was observed with increases in the maximum vasoactive–inotropic score (*p* < 0.001).

Although PELOD and PRISM scores were higher in the group with acute kidney injury, the difference was not statistically significant (*p* > 0.05). However, the risk of acute kidney injury was markedly increased as the RACHS-1 score increased (*p* < 0.001). Additionally, among intra-operative parameters, cardiopulmonary bypass duration, aortic cross-clamp time, and lactate levels at the time of admission to the ICU were significantly higher in the group with acute kidney injury (*p* < 0.05). A statistically significant difference was not observed between groups regarding the timing of the furosemide stress test and dosage of furosemide (mg/kg).

When the furosemide response was compared between the groups with and without acute kidney injury, the mean response was 9.86 ± 5.84 (median: 9.10) mL/kg/h in the group without acute kidney injury, while in the acute kidney injury group, the mean value significantly decreased to 5.07 ± 4.73 (median: 3.33) mL/kg/h (*t* = −5.765, *p* < 0.001). When the standardized fluid balance on the first postoperative day was compared between the groups with and without acute kidney injury, the mean in the group without acute kidney injury was −1 ± 1.85 mL/kg/h (median: −0.65 mL/kg/h), while the mean for the acute kidney injury group was significantly different at 1.33 ± 1.13 mL/kg/h (median: 1.21 mL/kg/h; *t* = −3.779, *p* < 0.001). While a significant difference was not observed between groups regarding their duration in the ICU or hospital stay, the acute kidney injury group had a significantly higher rate of mortality of 34% than the group without acute kidney injury (*p* < 0.001; Table 2).

In our study, when the timing of the first postoperative furosemide administration was evaluated, 23% of patients received furosemide within the first 6 h, 35% of patients received furosemide between 7 and 12 h and 42% of patients received furosemide between 13 and 24 h postoperatively.

To identify factors associated with the development of acute kidney injury (AKI), a multivariable logistic regression analysis was performed (Table 3). Age (*p* < 0.001), timing of the first furosemide administration (*p* = 0.005) and the first 2-h furosemide stress test response (*p* = 0.005) were inversely associated with AKI development. In contrast, lactate levels at ICU admission (*p* = 0.007) and first postoperative day fluid balance (*p* = 0.001) were identified as independent predictors associated with increased AKI risk. The RACHS score, vasoactive–inotropic score, and aortic cross-clamp time were not statistically significant in the adjusted model (*p* > 0.05). Model calibration assessed by the Hosmer–Lemeshow goodness-of-fit test demonstrated good agreement between observed and predicted outcomes (*p* > 0.05). No significant multicollinearity was detected, with variance inflation factor (VIF) values ranging between 1.00 and 1.73.

**Figure 1:**
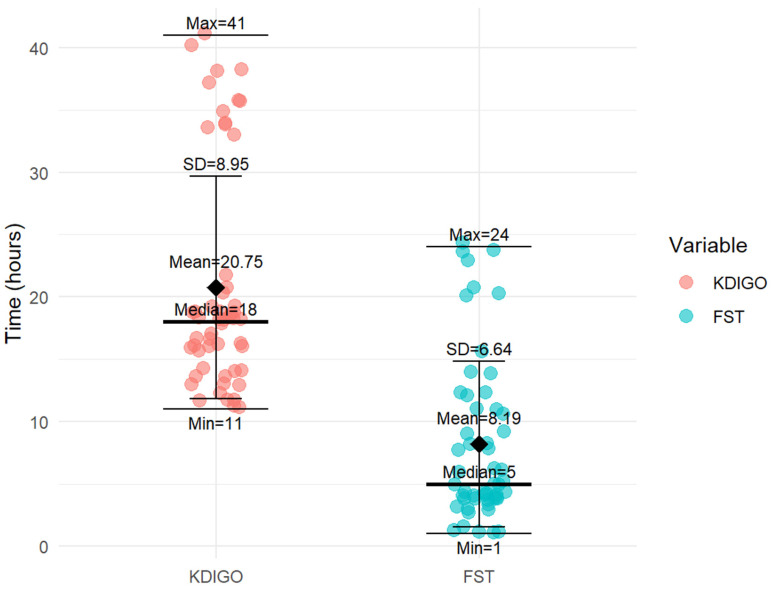
Earlier prediction of acute kidney injury with the furosemide stress test compared with KDIGO diagnostic criteria.

Acute kidney injury was diagnosed at a median of 18 h using the “Kidney Disease: Improving Global Outcomes” criteria, while the furosemide stress test enabled diagnosis at a median of 5 h (Figure 1).

Comparisons of patients who developed acute kidney injury according to the stages in the “Kidney Disease: Improving Global Outcomes” criteria are presented in Table 4. Although no significant differences were observed between the two groups in terms of age, body mass index, and sex (*p* > 0.05), the stages were found to increase significantly with higher PELOD and PRISM scores (*p* = 0.003). The duration of ICU stay was significantly longer for stages II and III compared to stage I (*p* = 0.013). While no mortality was observed for stage I, the mortality rate significantly increased with advancing stage, reaching 63.2% for stage III (*p* = 0.001). Similarly, the stage significantly increased with an increased rate of nephrotoxic medication use (*p* = 0.006). No other statistically significant differences between the groups were observed in the remaining parameters.

**Figure 2:**
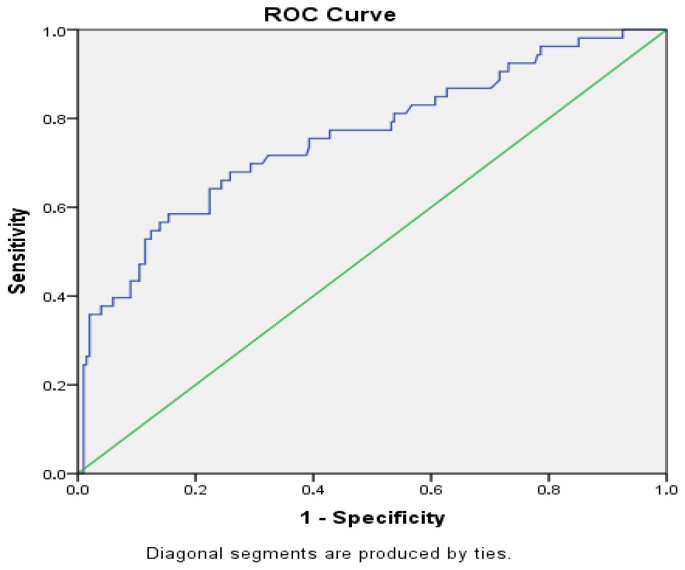
Receiver operating characteristic curve for prediction of acute kidney injury using the furosemide stress test. The blue line represents the ROC curve, and the green diagonal line represents the reference line indicating no discrimination (AUC = 0.5). blue line: ROC curve; green diagonal line: reference line representing no discrimination.

Receiver operating characteristic analysis was performed to assess the risk of the categorical variable “presence or absence of acute kidney injury” according to the variable for the “urinary response to furosemide stress test” (mL/kg/h) (Figure 2).

According to the results, the ability of the variable “urinary response to furosemide stress test” (mL/kg/h) to predict the development of acute kidney injury was calculated as an area under the curve of 0.757 (95% confidence interval: 0.679–0.836, *p* = 0.000) This value indicates that the furosemide stress test response has good discriminative ability for detecting the presence of acute kidney injury. The determined cut-off value was 6.104 mL/kg/h: patients below this threshold potentially have a higher risk of developing acute kidney injury. With this threshold, the sensitivity was 69.8%, and the specificity was 69.7% (Table 5).

## 4. Discussion

Early identification of acute kidney injury remains a major challenge in pediatric patients undergoing cardiac surgery, as traditional diagnostic criteria often delay recognition of renal dysfunction. The first 2-h urinary response following the furosemide stress test was evaluated. Similar studies assessing 6-h urine output after furosemide administration have shown that the maximum urine output occurs within the first 2 h. It has been reported that the first 2-h urine output after furosemide administration can be used to predict the risk of developing acute kidney injury [7,19]. A significantly lower urinary response to the furosemide stress test was observed in patients who developed acute kidney injury, suggesting that diminished diuretic response may reflect early tubular functional impairment. Accordingly, our primary finding indicates that early postoperative assessment of diuretic response following furosemide administration may help predict the risk of acute kidney injury in pediatric patients undergoing cardiac surgery (Table 2).

Although parenteral diuretics such as furosemide are widely used after pediatric cardiac surgery, there are very limited data describing the furosemide stress test in pediatric cardiac surgery. Given its cost-effectiveness and worldwide availability, we believe that furosemide could be used in the early detection of acute kidney injury rather than more expensive and less accessible novel biomarkers. Several studies have reported the better predictive ability of the furosemide stress test to determine acute kidney injury than serum and urinary biomarkers [9].

Frequent blood sampling is typically required for detecting and staging acute kidney injury in postoperative cardiac patients. Daily blood tests are obtained upon the patient’s initial arrival to the ICU and on subsequent days in our clinic and many other clinics worldwide. Furosemide is administered to all cardiac surgery patients in the early postoperative period once their hemodynamic status is stabilized. When fluid balance on the first postoperative day was compared between the groups in this study, patients who developed acute kidney injury demonstrated a more positive fluid balance. This finding suggests that increased fluid accumulation may be associated with impaired renal function and that fluid overload may play an important role in the development of acute kidney injury (Table 2).

Consistently, a few studies have also reported the early predictive value of the furosemide stress test for detecting acute kidney injury among patients during the postoperative period after cardiac surgery. Similar to our findings, patients who developed acute kidney injury in these studies also had a decreased urinary response to furosemide in the early postoperative period [7]. Low urinary response in the first 2 h after furosemide administration has been associated with adverse renal and clinical outcomes [7].

A separate study evaluating neonates and infants undergoing cardiac surgery also reported that diminished urine output in response to furosemide serves as a predictor of acute kidney injury development [19]. Neonates were not included in our study cohort because renal physiology, renal perfusion characteristics, and the pharmacokinetic and pharmacodynamic properties of furosemide during the neonatal period differ significantly from those of older infants and children. These developmental differences may result in variability in diuretic response and could limit the comparability of FST performance across age groups. Although age-stratified subgroup analyses were considered, it was anticipated that the sample size within each subgroup would be insufficient to provide adequate statistical power. Therefore, to establish a more homogeneous study population and to enhance the interpretability of the results, neonates were excluded from the study. Future multicenter prospective studies including neonatal populations are needed to better define the age-specific performance of the furosemide stress test.

In our study, diagnosis of acute kidney injury was made at a median of 18 h according to the “Kidney Disease: Improving Global Outcomes” criteria. Patients who developed acute kidney injury were administered the furosemide stress test at a median of postoperative 5 h and had a low urinary response (Figure 1). These findings suggest that the furosemide stress test can predict and detect acute kidney injury significantly earlier than kidney disease criteria, allowing early hemodynamic stabilization, discontinuation or adjustment of nephrotoxic medications, limitations to the progression of already existing acute kidney injury, and even potentially preventing its onset. A meta-analysis has shown that the furosemide stress test serves as a valuable and reliable biomarker for predicting the progression of acute kidney injury stage or the need for renal replacement therapy in the early stages of acute kidney injury development [20].

As this study had a retrospective design, the dose and timing of furosemide administration were not standardized. However, no statistically significant differences were observed between the acute kidney injury (AKI), and non-AKI groups in terms of furosemide dose (mg/kg) or the timing of administration during the furosemide stress test. When the timing of the first postoperative furosemide administration was examined, 23% of patients received furosemide within the first 6 h, 35% of patients received furosemide between 7 and 12 h, and 42% of patients received furosemide between 13 and 24 h. This distribution indicates that diuretic therapy following cardiac surgery should generally be individualized based on patients’ clinical and hemodynamic status rather than being applied according to a strict protocol. In the postoperative period, factors such as achieving hemodynamic stability, low cardiac output syndrome, or preload-dependent physiology may influence the timing of diuretic initiation. Therefore, variability in the timing of furosemide administration reflects real-world clinical practice. Despite this variability, the absence of significant differences between groups regarding dose and timing suggests that the predictive value of the furosemide stress test remains robust and is not substantially affected by these variations. Considering the fact that postoperative diuretic strategies may vary across different centres, the finding that the furosemide stress test remained a significant predictor of AKI despite variability in the timing of furosemide administration may support the generalizability of this method. Previous studies have also demonstrated that even with non-standardized furosemide dosing, the furosemide stress test retains strong discriminative ability in identifying the development of AKI [7]. Consistent with these findings, the significant predictive performance of the furosemide stress test in our cohort, despite variability in dosing and timing, supports its applicability in routine clinical practice. In another study that employed standardized furosemide dosing, initial postoperative furosemide administration was done within the first 8–24 h (median 12.8 IQR: 9.8–18.2), and the standard dosing was 0.8–1.2 mg/kg (mean: 0.98 mg/kg (standard deviation 0.06) [13]. The timing and dosing ranges were consistent with our study. Nevertheless, the lack of standardized dosing and timing should be considered an important limitation of this study. Future prospective studies using standardized protocols are warranted to more clearly define the predictive value of the furosemide stress test.

A reduced urinary response to the furosemide stress test demonstrated good discriminative ability for predicting acute kidney injury. A response below the identified cut-off value of 6.104 mL/kg/h was associated with a higher risk of acute kidney injury. The sensitivity and specificity of the test were approximately 70%, suggesting that the furosemide stress test should be considered not as a stand-alone diagnostic tool but rather as an early-risk stratification method. To date, only one pediatric study has reported a cut-off value for the FST response, identified as 3.6 mL/kg/h [7].

These findings suggest that the furosemide stress test may serve as an early warning and risk stratification tool in clinical practice. Patients with a reduced urinary response may benefit from closer renal and hemodynamic monitoring and early optimization of fluid and nephrotoxic exposure.

As secondary outcomes of our study, younger age, higher RACHS-1 and maximum vasoactive–inotropic score scores, longer aortic cross-clamp and cardiopulmonary bypass durations, and elevated lactate levels upon ICU admission were all associated with an increased risk of acute kidney injury (*p* < 0.005). Mortality was also significantly higher among patients who developed acute kidney injury (*p* < 0.005). Similarly, previous studies have reported that lower urine output at 2 h following furosemide administration is associated with prolonged ventilation time, sex, a higher Society of Thoracic Surgeons–European Association for Cardiothoracic Surgery (STAT) score, lower weight, younger age, and longer hospital and ICU stays [7]. The severity of the underlying cardiac disease, postoperative hemodynamic instability and non-pulsatile flow during cardiopulmonary bypass may contribute to decreased renal perfusion, thereby increasing the risk of kidney injury [21].

Multivariable logistic regression analysis demonstrated that age, timing of the first postoperative furosemide administration, the first 2-h furosemide stress test response, lactate levels at ICU admission, and fluid balance on the first postoperative day are independently associated with the development of acute kidney injury. The inverse association between early urinary response to the furosemide stress test and AKI suggests that FST may serve not only as a correlated marker but also as an independent predictor of AKI. In contrast, the RACHS score, vasoactive–inotropic score, and aortic cross-clamp time were not identified as independent risk factors in the adjusted model. These findings indicate that early functional renal assessment using the furosemide stress test, together with key clinical parameters, may facilitate early identification and risk stratification of pediatric cardiac surgery patients at increased risk for acute kidney injury.

In our cohort, the incidence of acute kidney injury following cardiac surgery was 20%, which is consistent with findings in the literature [3,4,5]. Patients who developed acute kidney injury in our study were grouped according to the “Kidney Disease: Improving Global Outcomes” criteria into mild, moderate, and severe stages. No demographic differences were observed among patients with varying severities of acute kidney injury. However, higher PRISM, PELOD, and RACHS-1 scores were associated with increased acute kidney injury severity, whereas no significant association was found between the maximum vasoactive–inotropic score and acute kidney injury stage. The percentage of patients who developed severe acute kidney injury according to the “Kidney Disease: Improving Global Outcomes” criteria (stages 2–3) was 15.6%. Similar studies have also reported similar percentages of patients with severe acute kidney injury (stages 2–3) within the range of 11.6–16% [1,2,3].

No significant association was found between the stage of acute kidney injury and the duration of cardiopulmonary bypass or aortic cross-clamp time in the patients who developed acute kidney injury in our study. While increasing the severity of acute kidney injury was associated with longer ICU stays, no differences were observed in overall hospital stay duration. Mortality rates increased substantially with higher acute kidney injury stages, reaching 63.2% in patients with stage-3 acute kidney injury. Greater exposure to nephrotoxic agents was also linked to higher acute kidney injury severity. Thus, these findings suggest that early detection of acute kidney injury may help prevent progression to more advanced stages.

Long-term outcomes at 6–12 months were evaluated in patients diagnosed with acute kidney injury according to the “Kidney Disease: Improving Global Outcomes” criteria. During follow-up, only two patients remained at acute kidney injury stage 1, and none of the patients required long-term renal replacement therapy. No association was found between the stage and persistent renal impairment, and (with the exception of only two cases) renal function normalized within 6–12 months.

**Limitations.** This study has several limitations. First, it was conducted at a single centre, and all the surgeries were performed by two different pediatric cardiac surgeons. Even though we were unable to assess whether patients received furosemide intra-operatively as well as the amount of intra-operative bleeding, we evaluated pre-furosemide hemodynamic stability (hemoglobin, central venous pressure, systolic blood pressure, and lactate levels), which may have partially mitigated this limitation. Additionally, as most patients received additional doses of furosemide within 6–12 h after the first dose, only the initial dose could be evaluated. Another limitation was that infants and children were not evaluated separately, despite having potential variability in pharmacokinetics of furosemide in different age groups. The recognition of acute kidney injury development using the furosemide stress test could not be compared with novel or other potential biomarkers due to the retrospective nature of our study. Finally, the retrospective study design could be a risk factor for data reliability, although all patient data were registered in the computer system simultaneously in the ICU and securely recorded in our hospital database system. Future prospective studies are needed to investigate the ability of the furosemide stress test to detect acute kidney injury in comparison with novel biomarkers and to validate our findings.

## 5. Conclusions

The findings of this study highlight the potential predictive value of the furosemide stress test for early identification of acute kidney injury in pediatric patients undergoing cardiac surgery. As an inexpensive and widely available tool, the furosemide stress test may facilitate early risk stratification and closer monitoring of high-risk patients, particularly in resource-limited settings. Prospective multicenter studies are warranted to further validate its predictive performance and define its role in clinical decision-making.

## Figures and Tables

**Table 1:** Demographic characteristics.

Descriptives	Number (%)
Sex	
Male	134 (52.8%)
Female	120 (47.2%)
Outcome	
Survive	236 (92.9%)
Mortality	18 (7.08%)
	Median (25th–75th percentile)
Age (months)	31 (11–64)
PELOD-2 Score *	3 (1–9)
PRISM-3 Score *	10 (7–18)
RACHS-1 Score *	2 (2–3)
Height (cm) *	90 (69–111)
Weight (kg) *	12 (7–18)
Length of Stay (days) *	
PICU	6.5 (4–13)
Hospital	12 (8–24)
Length of MV Support (Hours) *	31 (14–90)

* cm: centimetre, kg: kilogram, MV: mechanical ventilation, PELOD: Pediatric Logistic Organ Dysfunction, PICU: pediatric intensive care unit, PRISM: Pediatric Risk of Mortality, RACHS-1: Risk Adjustment for Congenital Heart Surgery-1.

**Table 2:** Comparison of patients with and without acute kidney injury.

Variable	Non-AKI	AKI	Test Statistic	*p* Value
Age (months)	50.47 ± 49.46 (39)	29.26 ± 38.60 (14)	Z = −3.147	<0.001
BMI *	15.38 ± 2.88 (15.03)	15.02 ± 3.09 (14.41)	Z = −1.497	0.134
Male sex, *n* (%)	104 (51.7)	30 (56.6)	χ^2^ = 0.398	0.528
PELOD score *	4.42 ± 6.06 (3)	16.58 ± 11.86 (21)	Z = −0.970	0.332
PRISM score *	10.38 ± 5.12 (10)	17.00 ± 5.09 (18)	Z = −0.584	0.559
RACHS-1 score *			χ^2^ = 42.243	<0.001
1	32 (15.9)	3 (5.7)		
2	98 (48.8)	14 (26.4)		
3	66 (32.8)	23 (43.4)		
4	5 (2.5)	7 (13.2)		
5	0 (0)	2 (3.8)		
6	0 (0)	4 (7.5)		
VIS score *			χ^2^ = 54.113	<0.001
0	17 (8.5)	1 (1.9)		
1	1 (0.5)	0 (0)		
2	92 (45.8)	5 (9.4)		
3	76 (37.8)	24 (45.3)		
4	15 (7.5)	23 (43.4)		
Cardiopulmonary bypass duration (min)	123.9 ± 67.27 (111)	165.43 ± 85.71 (162)	Z = −0.412	0.680
Aortic clamp duration (min)	72.65 ± 45.25 (61)	86.33 ± 47.06 (79)	Z = −2.033	0.042
Lactate level at ICU admission (mmol/L) *	3.38 ± 1.97 (2.80)	5.35 ± 3.03 (4.75)	Z = −5.749	<0.010
First dose of furosemide (mg/kg)	0.5 ± 0.18 (0.5)	0.88 ± 0.46 (1)	Z = −0.252	0.802
Timing of the first dose of furosemide (postoperative hour)	12.37 ± 3.47 (12)	8.15 ± 2.52 (5)	Z = −1.128	0.259
Urine output in the first 2 h after furosemide (mL/kg/h)	9.86 ± 5.84 (9.10)	5.07 ± 4.73 (3.33)	Z = −5.765	<0.001
Postoperative day-1 fluid balance (mL/kg/h)	−1.00 ± 1.85 (−0.65)	1.33 ± 1.13 (1.21)	Z = −3.779	<0.001
ICU stay duration (days) *	7.46 ± 9.41 (5)	48.41 ± 68.66 (27)	Z = −0.831	0.406
Duration of hospital stay (days)	14.86 ± 13.9 (10)	62.67 ± 75.60 (30)	Z = 1.195	0.232
Mortality, *n* (%)	0 (0)	18 (34)	χ^2^ = 72.78	<0.001

Data are presented as mean ± SD (median) or *n* (%). Comparisons between groups were performed using the Mann–Whitney U test or Chi-square test, as appropriate. Statistically significant *p* values (*p* < 0.05) are shown in bold. * AKI: acute kidney injury; BMI: body mass index; ICU: intensive care unit; PELOD: Pediatric Logistic Organ Dysfunction; PRISM: Pediatric Risk of Mortality; VIS: vasoactive–inotropic score; RACHS: Risk Adjustment for Congenital Heart Surgery.

**Table 3:** Multivariable logistic regression analysis for predictors of postoperative AKI.

Variable	β Coefficient	Std. Error	*z* Value	*p* Value	Odds Ratio (OR)	95% CI for OR
Furosemide timing (h)	−0.110	0.039	−2.807	0.005	0.90	0.83–0.97
Age (month)	−0.030	0.008	−3.620	<0.001	0.97	0.95–0.99
RACHS score	0.273	0.310	0.879	0.379	1.31	0.71–2.41
VIS score	0.550	0.317	1.734	0.083	1.73	0.93–3.21
Aortic clamp time (min)	0.00004	0.005	0.009	0.993	1.00	0.99–1.01
Lactate level (ICU admission) (mmol/L)	0.307	0.114	2.689	0.007	1.36	1.09–1.69
FST response (first 2 h)	−0.140	0.050	−2.802	0.005	0.87	0.79–0.96
First-day fluid balance (mL)	0.0037	0.0011	3.277	0.001	1.004	1.002–1.006

VIS: vasoactive–inotrope score; FST: furosemide stress test; min: minute; h: hour; mL: millilitre.

**Table 4:** Comparison of patients with acute kidney injury according to KDIGO stages.

Variable	KDIGO I	KDIGO II	KDIGO III	Test Statistic	*p* Value
Age (months)	38.07 ± 53.0 (16)	30.57 ± 40.86 (14)	21.78 ± 21.43 (9)	KW = 0.145	0.930
BMI *	15.35 ± 4.28 (14.84)	15.32 ± 2.75 (14.57)	14.47 ± 2.53 (14.19)	KW = 1.274	0.529
Male sex, *n* (%)	8 (61.5)	10 (52.4)	11 (57.9)	χ^2^ = 0.294	0.863
Female sex, *n* (%)	5 (38.5)	11 (47.6)	8 (42.1)		
PELOD score *	9.46 ± 9.76 (3)	14.52 ± 12.01 (11)	23.73 ± 9.33 (24)	KW = 11.552	0.003
PRISM score *	14.15 ± 4.74 (17)	16.04 ± 5.69 (18)	20.00 ± 2.72 (18)	KW = 11.862	0.003
RACHS-1 score, *n* (%) *				χ^2^ = 17.624	0.062
1	0	3 (14.3)	0		
2	5 (38.5)	6 (28.6)	3 (15.8)		
3	5 (38.5)	7 (33.3)	11 (57.9)		
4	1 (7.7)	4 (19.0)	2 (10.5)		
5	2 (15.4)	0	0		
6	0	1 (4.8)	3 (15.8)		
VIS, n (%) *				χ^2^ = 8.320	0.216
0	0	1 (4.8)	0		
1	0	0	0		
2	3 (23.1)	2 (9.5)	0		
3	7 (53.8)	9 (42.9)	8 (42.1)		
4	10 (23.1)	9 (42.9)	11 (57.9)		
CPB duration (minute) *	140.76 ± 64.20 (160)	148.66 ± 69.87 (162)	200.84 ± 104.69 (183)	KW = 3.347	0.188
Aortic cross-clamp duration (minute)	78.30 ± 40.47 (95)	85.09 ± 46.39 (76)	93.21 ± 53.08 (79)	KW = 0.225	0.893
First postoperative day fluid balance (mL)	282.84 ± 327.58 (139)	266.66 ± 310.15 (156)	311.73 ± 337.48 (169)	KW = 0.232	0.891
ICU stay duration (days) *	16.30 ± 13.80 (12)	65.47 ± 96.48 (28)	51.52 ± 45.36 (30)	KW = 8.643	0.013
Hospital stay (days)	32.00 ± 24.47 (22)	76.76 ± 100.35 (30)	68.10 ± 63.11 (49)	KW = 3.833	0.147
Number of nephrotoxic drugs, *n* (%)				χ^2^ = 18.092	0.006
0	10 (76.9)	9 (42.9)	2 (10.5)		
1	3 (23.1)	5 (23.8)	12 (63.2)		
2	0	5 (23.8)	3 (15.8)		
≥3	0	2 (9.5)	2 (10.5)		
6th month creatinine (mg/dL)	0.36 ± 0.19 (0.30)	0.32 ± 0.10 (0.30)	0.29 ± 0.07 (0.30)	KW = 0.350	0.839
6th month KDIGO stage, *n* (%) *				χ^2^ = 0.540	0.764
0	12 (92.3)	14 (93.3)	7 (100)		
1	1 (7.7)	1 (6.7)	0		
Mortality present *n* (%)	0 (0)	6 (28.6)	12 (63.2)	χ^2^ = 14.179	<0.001

Data are presented as mean ± SD (median) or *n* (%). Comparisons among KDIGO stages were performed using the Kruskal–Wallis test or Chi-square test, as appropriate. Statistically significant *p* values (*p* < 0.05) are shown in bold. * KDIGO: Kidney Disease: Improving Global Outcomes; BMI: body mass index; ICU: intensive care unit; CPB: cardiopulmonary bypass; PELOD: Pediatric Logistic Organ Dysfunction; PRISM: Pediatric Risk of Mortality; VIS: vasoactive–inotropic score; RACHS: Risk Adjustment for Congenital Heart Surgery.

**Table 5:** Predictive performance of the furosemide stress test for acute kidney injury.

Risk Factor	AUC (95% CI)	Cut-Off (mL)	*p* Value	Sensitivity	Specificity
Furosemide stress test urinary response	0.757 (0.679–0.836)	6.104	<0.001	0.698	0.697

AUC, area under the curve; CI, confidence interval.

## Data Availability

The datasets used and/or analyzed during the current study are available from the corresponding author on reasonable request.

## Abbreviations

The following abbreviations are used in this manuscript:

FST	Furosemide stress test
AKI	Acute kidney injury
KDIGO	Kidney Disease: Improving Global Outcomes
ICU	Intensive care unit
CPB	Cardiopulmonary bypass
PELOD-2 scores	Pediatric Logistic Organ Dysfunction-2
PRISM-III scores	Pediatric Mortality Risk-3
RACHS-1 scores	Risk Adjustment for Congenital Heart Surgery-1
VIS	Vasoactive–inotropic score
ROC	Receiver operating characteristics
AUC	Area under curve
CI	Confidence interval
